# Supplementary material for: Patterns of Intron Gain and Loss in Fungi
Source: PLoS Biol. 2004 Nov 30;2(12):e422. doi: 10.1371/journal.pbio.0020422 (PMC532390; doi:10.1371/journal.pbio.0020422)
Supplement: Table S1 — Also available at http://genes.mit.edu/NielsenEtAl/. (4.3 MB ZIP). [file pbio.0020422.st001.zip › NielsenEtAl/html/404.html]

AN0918.1.NCU03492.1.MG03920.1.FG05164.1


```
 CLUSTAL W (1.82) Multiple Sequence Alignments - Introns Inserted


Sequence 1: NCU03492.1	371 aa
Sequence 2: MG03920.1	384 aa
Sequence 3: FG05164.1	379 aa
Sequence 4: AN0918.1	362 aa
Alignment Length: 391 aa
Number Identitical Residues: 192 aa
Alignment Score (without introns) 10360


MG03920.1 	MPSLTLPSFTQAEVEAHNTNRSCYVTVGQNVYDVTDFLDAHPGGADLVLDYAGKDIGAIL
NCU03492.1	MSGRTLPTLTIDEVESHNSAKSCYVTVGRNVYDVTDFLDSHPGGADLVLDWAGKDIGAIL
FG05164.1 	MPGLTLPTFTRAEVEEHNSEGSCYVTIGSKVYDITDFIEDHPGGPEYVLEYAGRDIEKIL
AN0918.1  	MPGQTLPTFTPAEVESHNSAKSCYVTIGSKVYDITSFVDDHPGGGDLVLEYAGKDVTEIL
          	*.. ***::*  *** **:  *****:* :***:*.*:: **** : **::**:*:  **

MG03920.1 	KDEDSHTHTEAAYEILEESLVGFLKPDKSF---SNGKANGNGAAKGSSSGVDTPELVDAQ
NCU03492.1	KDEASHTHSEAAYEVLDESLVGYLAS-------GNG-AAANGTTKDGENGTEQ-------
FG05164.1 	KDSDSHTHSDSAYEILDESLVGFLVPEKSVNGHANGTANGNGNAKLSNGDANG-------
AN0918.1  	RDPVSHTHSESAYEILEDNLVGFIGS--------------ESSSKCANGSANG-------
          	:*  ****:::***:*::.***:: .              :. :* .....:        

MG03920.1 	GRPVHPRTGMSCEEDLNKETDYTGDYKKHKFLDLNKPLLMQVFLGGFSKEFYLEQVHRPR
NCU03492.1	-QWVHPRTGMSCEEDLSKDTDYNADYKTHKFLDLNKPLFMQIWNGGFSKEFYLDQVHRPR
FG05164.1 	--TVHPRTGMSCEEDLSRETDYNMDYKKNKFLDLNRPLFPQLWFGGFTKEFYLDQVHRPR
AN0918.1  	-KPVYASTGMSTAEDLSVETDAVQDYQKHKFLDLNKPLLMQLWNSGFSKEFYLEQVHRPR
          	   *:. ****  ***. :**   **:.:******:**: *:: .**:*****:******

MG03920.1 	HYKGGESAPLFGNFLEPLSKTAWWVIPIVWLPPVAYGTYLASQGIESKLNLAALWCSGIA
NCU03492.1	HYKGGESAPLFGNFLEPLSKTPWWLIPLVWWPPISYGVSVAFQGFGSVAPVAGYFGFGLA
FG05164.1 	HYKGGQSAPLFGNFLEPLSKTAWYIVPIIWLPCIAFGTHVASQGFDNQLYTLGYWVFGVF
AN0918.1  	HYKGGDSAPLFGNFLEPLSKTAWYVVPIVWLPPVLYGTYLGASGLGRAPAAAAYWLFGFF
          	*****:***************.*:::*::* * : :*. :. .*:       . :  *. 

MG03920.1 	LWTIIEYILHR~FLFHLDK2YLPDNRFAITLHFLLHGVHHYLPMDKLRLVMPPTLFFVLA
NCU03492.1	FWTLIEYILHR~FLFHLDY2YLPDNRVGITAHFLLHGIHHYLPMDRYRLVMPPTLFVVLA
FG05164.1 	FWTIIEYVLHR~FLFHLDY2YLPDNRVGITLHFVLHGIHHYLPMDRYRLVMPPALFALLA
AN0918.1  	LWSLIEYLMHR2-------~YLPDNRVGITLHFLLHGIHHYLPMDKYRLVMPPSLFVILA
          	:*::***::**         ******..** **:***:*******: ******:** :**

MG03920.1 	FPFWKLAHFLFWYNWHVGTAIFCGGIFGYICYDLTHYFLHHKN~LPLWYKDLKKYHLEHH
NCU03492.1	APFWKFAHAILFYNWYAATAAFCGGVFGYTLYDMTHYFLHHQN~LPAYYKELKKYHLAHH
FG05164.1 	TPFWWIAHSILCHNWYAAVAAYCGGVFGYVCYDLTHYFLHHQD~LPLWYKDLKKYHLAHH
AN0918.1  	TPFWKLAHTVFYYNWNAAVLAYCGGVFGYICYDLTHYFLHHRN2LPSYYKGLKKYHLEHH
          	 *** :** :: :** ...  :***:***  **:*******:: ** :** ****** **

MG03920.1 	FLDYENGFGVTSRFWDRVFGTEIVPLVKTK----
NCU03492.1	FLDYENGFGVTSAFWDKIFGTELVYNPKTN----
FG05164.1 	FLDYELGFGVTSRFWDTIFGTELIYDTKKTK---
AN0918.1  	FADYDNGFGVTSRFWDWVFGTELELPPPKVLKTQ
          	* **: ****** *** :****:     .  .:.
```
